# Effects of reproduction and environmental factors on body temperature and activity patterns of wolverines

**DOI:** 10.1186/s12983-019-0319-8

**Published:** 2019-06-17

**Authors:** Alexandra Thiel, Alina L. Evans, Boris Fuchs, Jon M. Arnemo, Malin Aronsson, Jens Persson

**Affiliations:** 1 Department of Forestry and Wildlife Management, Inland Norway University of Applied Sciences, Campus Evenstad, NO - 2480 Koppang, Norway; 2 Department of Wildlife, Fish and Environmental Studies, Swedish University of Agricultural Sciences, Umeå, Sweden; 3 Department of Zoology, Stockholm University, Stockholm, Sweden; 4 Department of Ecology, Grimsö Wildlife Research Station, Swedish University of Agricultural Sciences, Riddarhyttan, Sweden

**Keywords:** Biologging, Circadian rhythm, Ecophysiology, Gestation, Heterothermy, Northern ecosystem, Seasonality

## Abstract

**Background:**

Mammals in the far north are exposed to extreme seasonal changes in environmental conditions, such as temperature and photoperiod, which have notable effects on animal physiology and behaviour. The wolverine (*Gulo gulo*) is a carnivore with a circumpolar distribution and well-adapted to extreme environmental conditions. Still, ecophysiological studies on free-ranging wolverines are lacking. In this study, we used abdominally implanted body temperature loggers in combination with GPS collars with acceleration sensors on 14 free-ranging wolverines in northern Sweden to study daily and seasonal variation in body temperature and activity patterns. We used generalized additive mixed modelling to investigate body temperature patterns over time and Lomb-Scargle periodogram analysis to analyse circadian rhythms.

**Results:**

We found that wolverines have an average core body temperature of 38.5 ± 0.2 °C with a daily variation of up to 6 °C. Body temperature patterns varied between reproductive states. Pregnant females showed a distinct decrease in body temperature during gestation. Wolverines were active both in day and night, but displayed distinct activity peaks during crepuscular hours. However, body temperature and activity patterns changed seasonally, with a gradual change from a unimodal pattern in winter with concentrated activity during the short period of day light to a bimodal pattern in autumn with activity peaks around dusk and dawn. Wolverines were less likely to display 24-h rhythms in winter, when hours of day light are limited.

**Conclusions:**

The combination of different biologging techniques gave novel insight into the ecophysiology, activity patterns and reproductive biology of free-ranging wolverines, adding important knowledge to our understanding of animals adapted to cold environments at northern latitudes.

**Electronic supplementary material:**

The online version of this article (10.1186/s12983-019-0319-8) contains supplementary material, which is available to authorized users.

## Background

Knowledge of an animal’s body temperature (hereafter: T_b_) profile provides information on thermoregulation, physiology and behaviour, as well as insight into animals` response to changing environmental conditions [1]. Despite large thermal fluctuations, endothermic species are able to regulate T_b_ within a species-specific narrow range and hence maintain relatively stable core T_b_ (homeothermy). In mammals, species-specific normal T_b_ varies from 30 to 40 °C [2] and often displays daily oscillations of 1–4 °C [3]. However, the greatest daily amplitude reported for a free-living large mammal that does not use torpor (Aardvark [*Orycteropus afer*]) was 8.6 °C, during reduced prey availability [4]. Changing environmental conditions not only impact seasonal and daily patterns of T_b_ in mammals [5–8] but also the circadian rhythmicity of activity [9]. Circadian rhythms are daily oscillations, which are at least partly endogenously controlled and recur in approximately 24-h intervals [10]. For instance, several species in the far north lack circadian rhythmicity in activity in summer, when the sun never sets [11, 12], indicating the importance of the light-dark cycle as a *Zeitgeber* to entrain circadian rhythms. Still, Arctic ground squirrels (*Spermophilus parryii)* maintain circadian rhythms under constant light conditions [13]. Studies of animals in laboratory conditions suggest that daily rhythms in T_b_ and activity are highly correlated [14–16], but this relationship is largely unexplored in free-ranging animals (but see [17, 18]). Studies in laboratory conditions have also found a profound influence of pregnancy on the physiology of mammals and T_b_ in particular [19–21]. This is expected to be present under natural conditions as well. For instance, pregnant female lions (*Panthera leo*) exhibit gestational hypothermia and decrease their T_b_ by 1.3 °C during gestation [22].

Species that are adapted to cold climates, such as the wolverine *(Gulo gulo*; [23–25]), may be particularly vulnerable to rising ambient temperatures associated with global warming [25–27]. The need for a framework incorporating ecology and physiology of cold-adapted mammals to identify vulnerability to winter climate change has been emphasized [28], as well as a need for long-term measurements of physiology of mammals in cold environments [29]. Current information on wolverine thermal physiology is limited to a single study of 4 captive individuals, where T_b_ was measured 4 times a day for 82 days during constant daylight and darkness [30]. Thus, information on year-round variation of wolverine T_b_ in their natural environment and light conditions is lacking. While wolverine reproductive biology has been studied in harvested [31], captive [32] and radio-collared animals [33–36], information on physiological characteristics during reproduction is lacking. Female wolverines display embryonic diapause [31, 37] after mating (April – August) [37] with implantation occurring from December to February. Literature suggests a gestation period of 30–50 days [32] and peak parturition from February to mid–March [36], which is earlier than for other northern non-hibernating carnivores [25].

**Fig. 1:**
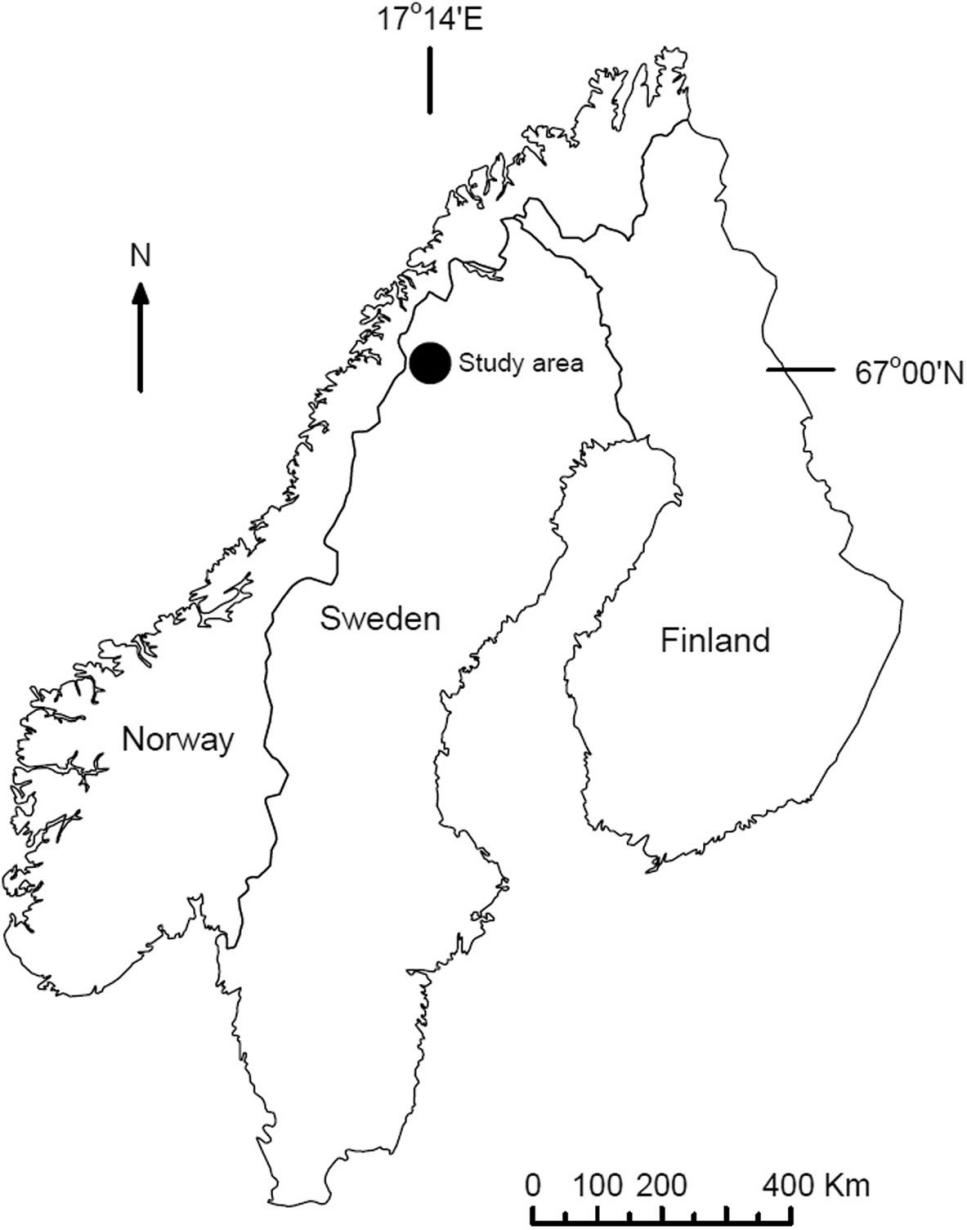
The location of the study area in northern Sweden where wolverines where monitored from 2011 to 2014

In this study, we used intraperitoneally implanted T_b_ loggers to record core T_b_ in wolverines in northern Sweden (Fig. 1). In addition to T_b_ loggers, wolverines were equipped with global positioning system (GPS) collars with acceleration sensors to record locomotor activity. The combination of biotelemetry and biologging helps to link individual behaviour with physiology and energy status, while recognizing individual variation. This provides key information for conservation efforts of endangered species that are difficult to study with conventional methods [38] and are affected by human activities [39], such as the wolverine is in Sweden [40]. Wikelski and Cooke [41] proposed that conservation strategies would benefit from a better understanding of the physiological responses of organisms to a changing environment.

**Fig. 2:**
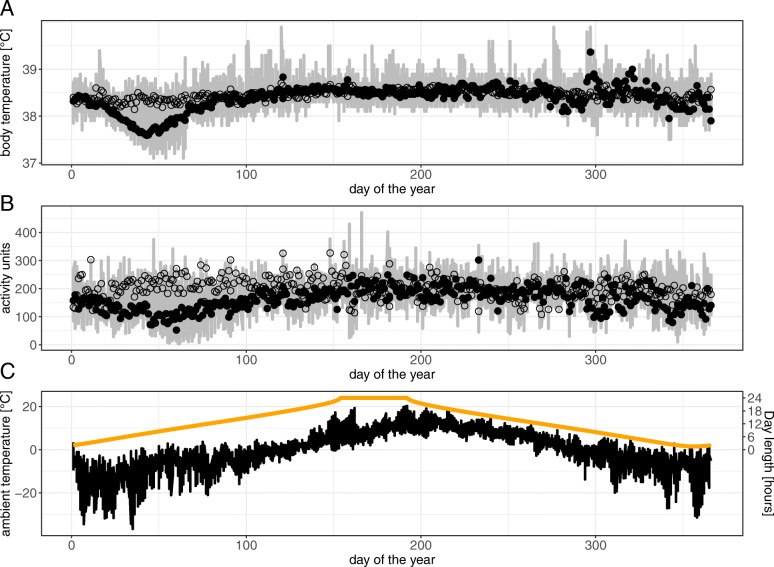
Daily averaged (**a**) body temperature (T_b_, [°C]) and (**b**) activity of each individual wolverine (grey line) and all individuals together (pregnant individuals: black dots, non-pregnant individuals: hollow circles) and (**c**) daily averaged ambient temperature from 2011 to 2014 [°C] (black lines) and day length [hours] (orange line)

The objectives of this study were to describe seasonal changes in wolverine T_b_ and to explore how reproductive state and environmental conditions influence T_b_, as well as activity. We hypothesize that wolverines will lose circadian rhythmicity in T_b_ and activity in arctic winter and summer. Our aim was to increase the understanding of physiological and behavioural characteristics of a species that is considered highly adapted to cold climate (Fig. 2c).

## Methods

### Study area

The study area encompasses approximately 7000 km^2^ and is located in and around the Laponia UNESCO World Heritage site (Kvikkjokk 67°00′N, 17°40′E, Fig. 1). The area is characterized by deep valleys at about 300 m above sea level (m.a.s.l.) and high mountainous plateaus of bare rock and glaciers with peaks up to 2000 m a.s.l. Tundra represents 57% of the study area and forest 36% [42]. Valleys are dominated by mountain birch (*Betula pubescens*), Scots pine (*Pinus sylvestris*), and Norway spruce (*Picea abies*), while mountain birch forms the tree line at 600–700 m a.s.l [43]. The climate is continental with distinct seasons and the ground is usually snow-covered from November to late May, but with large altitudinal variation. Monthly mean ambient temperatures range from − 12 °C in January to 12 °C in July (www.smhi.se; weather station Mierkenis; 66°68′ N, 16°6′E) and minimum day length (1.5 h) can be detected in December, whereas maximum day length of 24 h can be seen when the sun never sets from end of May until mid-July.

### Data collection

Adult wolverines were captured by darting from helicopter in May, June and October 2011–2014. Juveniles were located by radiotracking the mother and subsequently captured on the ground [34, 44]. For individuals not captured as juveniles (i.e. of known age, *n* = 5), approximate age was estimated based on reproductive history [35] and/or genetic relatedness to individuals with known age [45]. Animals were handled, implanted with T_b_ loggers (T_b_ loggers: DST Centi, Star Oddi, Gardabaer, Iceland, 46 × 15 mm, 19 g) and VHF abdominal implants (IMP/150/L [21 g] and IMP/400/L [~ 95 g], Telonics, Mesa, Arizona, USA), and adults were fitted with GPS collars (Vectronics-aerospace, Berlin, Germany) according to established protocols [44, 46]. Adult wolverines were darted from helicopter with 4 mg medetomidine (Zalopine® or Domitor®, Orion Pharma Animal Health, Turku, Finland) + 100 mg ketamine (Narketan®, Chassot, Dublin, Irleand). Juveniles were immobilized with 0.1 mg/kg medetomidine + 5 mg/kg ketamine (i.m.). For further information on surgical procedures see Additional file 4. T_b_ loggers were individually calibrated by the manufacturer over the range of 5 °C to 45 °C and recorded T_b_ with a guaranteed accuracy of ±0.1 °C 1 year post calibration. Loggers were programmed to record T_b_ every 5, 10 or 15 min. All GPS collars were equipped with activity sensors measuring acceleration continuously 6–8 times per second in 2 orthogonal directions (X and Y axis). Acceleration values were averaged for each direction for a recording interval of 5 min, ranging from 0 to 255 for each axis and the sum was used to present overall activity, resulting in values from 0 to 510 [47]. Animals were recaptured in order to retrieve T_b_ loggers and GPS collars, and to downloaded data. In total, T_b_ data were obtained from 14 wolverines (13 females and 1 male; 4 months to 10 years old), after 2–24 months of data collection. For 10 of these wolverines, activity data was obtained for the same time period as T_b_ (9 females and 1 male). Data from the week of capture and fever events (daily mean T_b_ > = 40 °C) were excluded from all analyses. Three of the animals were found dead (2–16 months after capture) and data from the day of death were excluded. We identified 4 occasions in 4 individuals when when T_b_ dropped to unrealistic low T_b_ values (< 34 °C), followed by immediate return to baseline values, indicating T_b_ logger failure. These observations were identified and replaced by the average of the 3 previous and subsequent observations. Data for these four individuals were therefore included in analysis of daily mean T_b_ but days, with replaced data were excluded from analyses with daily variation as response variable. In total, we used 1,027,345 T_b_ measurements from 14 wolverines in 32 individual years and 922,673 activity measurements from 10 wolverines in 18 individual years.

### Reproductive state

Female reproductive state was defined as either pregnant or non-pregnant based on daily mean T_b_. Females were defined as pregnant if they showed a distinct pattern of continuously decreasing T_b_ in the beginning of the year (Additional file 1). This distinct pattern of decreasing T_b_ during gestation was evident in at least one breeding season for all 8 females older than 2 years, and in 10 out of 13 (77%) individual breeding seasons. Denning was documented subsequent to these 10 pregnancies in 4 cases and cubs were observed in June in 3 cases (J. Persson, unpublished data). Pregnancy was not detected in any of the 2 years-old females, although females can reproduce at this age [35]. The date of implantation was determined as the day when the trend of decreasing T_b_ began, using changepoint analysis for daily mean T_b_ in the R package ‘*changepoint*’ [48]. This method detects multiple change points in a time series using a pruned exact linear time (PELT) algorithm, which identifies the maximum number of segments a time series can be split into [49], and has been applied for changes in T_b_ [50]. Date of parturition was determined as the day when mean T_b_ showed a sudden increase (Additional file 1), resulting in values comparable to normal T_b_, reached within a few days. Additionally, activity and location data from GPS collars were used to assess date of parturition. It was determined as the day in which daily mean activity showed a sudden decrease (Additional file 1) followed by low values over several days, and when location data showed that a female remained in a very restricted area for several consecutive days. For non-pregnant females, corresponding gestation period was determined as the period between the average date of implantation and parturition of pregnant females.

### Statistical analysis

To investigate the annual variation in wolverine T_b_ and test for differences between reproductive states throughout the year we fitted Generalized additive mixed models (GAMM) with the function “*bam*” from the R package “*mgcv*” [51, 52]. GAMMs are particularly suitable for handling non-linear relationships between response and predictor and handling autocorrelation [53]. We used daily mean T_b_ as response variable, added a fitted smooth term for day of the year (DOY, 1–366), an ordered factor for reproductive state (pregnant vs. non-pregnant) and an ordered-factor-smooth-interaction term for day of the year multiplied by reproductive state. We included a random intercept and slope for each wolverine ID and added an autoregressive model (AR1) structure to account for detected residual temporal autocorrelation. We applied the “*gam.check*” function of the “*mgcv*” package to choose adequate basis dimension of *k* [51]. The difference of daily mean T_b_ between reproductive states was considered significant on days when the 95% confidence interval of the modelled difference did not overlap zero.

To investigate annual heterothermy we calculated the daily variation in T_b_, defined as the individual range in wolverine T_b_ (individual daily maximum T_b_ – minimum T_b_). We fitted GAMMs on the individual daily variation in T_b_ as response variable with the same combination of smooth terms, random structure and AR1 structure as in the model for annual variation in daily mean T_b_.

To investigate the daily relationship between T_b_, activity, photoperiod and ambient temperature (T_a_) graphically, we averaged these parameters hourly for every individual, but excluded pregnant females during the time of gestation. We used a gam smoother with cubic spline function in the R package *ggplot* [54] on these hourly values averaged over each season to interpret visually how daily patterns in T_b_ and activity co-vary with T_a_ and photoperiod. Seasons were defined according to the Swedish Meteorological and Hydrological Institute (SMHI) based on ambient temperature climate indicator as the following: Winter (December – February), Spring (March – May), Summer (June – August), Autumn (September– November) [55]. Hourly T_a_ data were obtained from the SMHI weather station in Mierkenis, Sweden. Sunrise, sunset, dawn, dusk and day length for the study area were calculated using the R packages "*rgeos*" and "*maptools*" [56, 57] and defined as followed: sunrise and sunset are the points at which the top edge of the sun reaches the horizon (i.e. when the top of the sun < 1° below the horizon), dusk and dawn were defined as the onset and end of civil twilight (i.e. when the sun is 6° below the horizon in the morning and evening, respectively).

To investigate periodicity of circadian rhythms in T_b_ and activity we used Lomb-Scargle periodogram analysis in the R package "*lomb*" [58]. This method can detect periodicity in irregular spaced time series with missing values [58]. Analyses were performed on the raw data on a 15-day rolling window, i.e. we defined the periods by the DOY, selected 7 days on either side, performed the analysis and moved one DOY ahead. We tested for presence and significance of rhythms between 2 and 30 h, selected the highest significant peak and rounded it to the full hour. Significant periodicity was recognized when peaks in the Lomb-Scargle periodogram exceeded the 95% confidence limit. We determined the two most frequent period lengths (24 or 12 h), displayed by wolverines over the year and converted them into a binomial variable with 1 representing an approximate 24 or 12 h rhythm that day and 0 representing lack of a 24 or 12 h rhythm. To investigate if the probability of wolverines displaying a 24 or 12 h rhythm in T_b_ and activity changes over the course of a year, we fitted separate binomial GAMMs on the presence of the rhythm in question (binomial variable) as response variable. We added a smooth term for DOY and a random intercept and slope for each wolverine ID. We checked for autocorrelation in each model and decided that it was in an acceptable range and including an autocorrelation structure not necessary (Additional file 3).

Means are presented with associated standard deviation (mean ± SD) in the following text, if not otherwise stated.

## Results

### Annual variation in body temperature

On an annual basis, daily mean T_b_ for non-pregnant females (*n* = 6) was 38.5 ± 0.2 °C, and the range of individual daily variation was 0.9–6.0 °C (mean: 2.5 ± 0.7 °C). A steady decrease in daily mean T_b_, indicating gestational hypothermia (Fig. 2a), revealed 10 pregnancies for 8 females > 2 years old and that all adult females were pregnant at least once. These 8 females were considered pregnant during 10 individual breeding seasons in further statistical analysis. Pregnancy was not revealed in either of the 2 – year old females (*n* = 2).

**Fig. 3:**
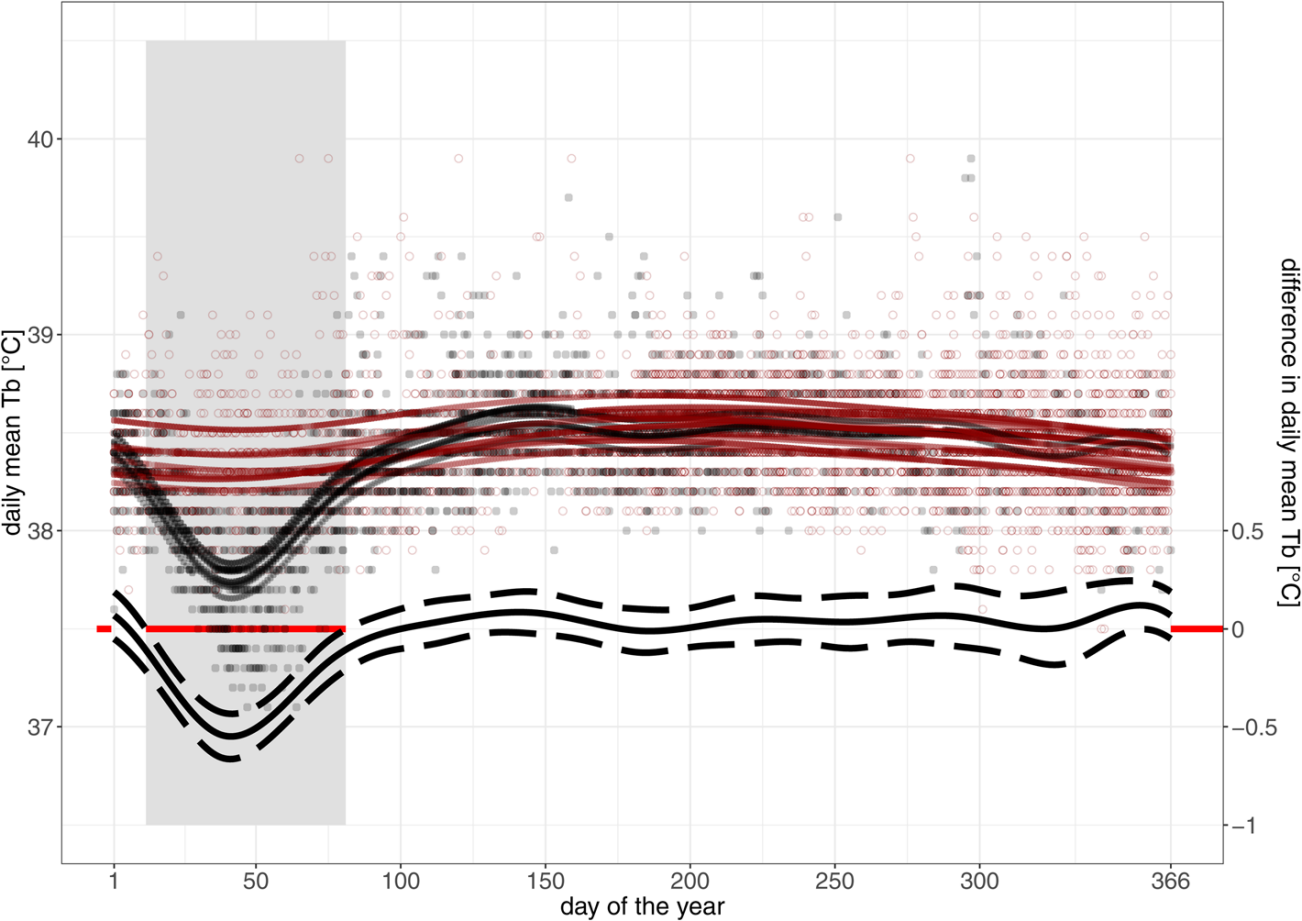
Daily mean body temperature (T_b_) [°C] for eight pregnant (black hollow circles) and six non-pregnant wolverines (red hollow circles) over the year (day of the year 1 = 1st January). Fitted values from GAMM model are presented for pregnant (black lines) and non-pregnant individuals (red lines). On the bottom, the black line shows the average modelled difference in daily mean T_b_ [°C] with 95% CI (dashed lines) between pregnant and non-pregnant wolverines, with a red line at 0 °C difference in the background as a reference level. The grey ribbon in the background shows the time span in which T_b_ of pregnant females is significantly different from T_b_ of non-pregnant wolverines

Non-pregnant wolverines showed a non-linear trend of increasing and decreasing daily mean T_b_ over the year. Lowest predicted values were found on day (DOY) 42 with 38.3 °C (95% CI: 38.2–38.4 °C), followed by an increase with predicted peak T_b_ on DOY 196 with 38.6 °C (95% CI: 38.5–38.7 °C) and a subsequent decrease towards the end of the year (Fig. 3). Daily mean T_b_ of pregnant females (*n* = 8) was significantly lower than for non-pregnant females from DOY 12–81 (i.e. modelled maximum difference of − 0.5 °C [95% CI: − 0.4 – − 0.6 °C] on DOY 41) but did not differ for the rest of the year (Fig. 3). At the day before parturition, pregnant females had a daily mean T_b_ of 37.5 ± 0.2 °C, which presented a decrease by 0.8 ± 0.3 °C when compared to T_b_ in December.

### Daily variation in body temperature and activity

**Fig. 4:**
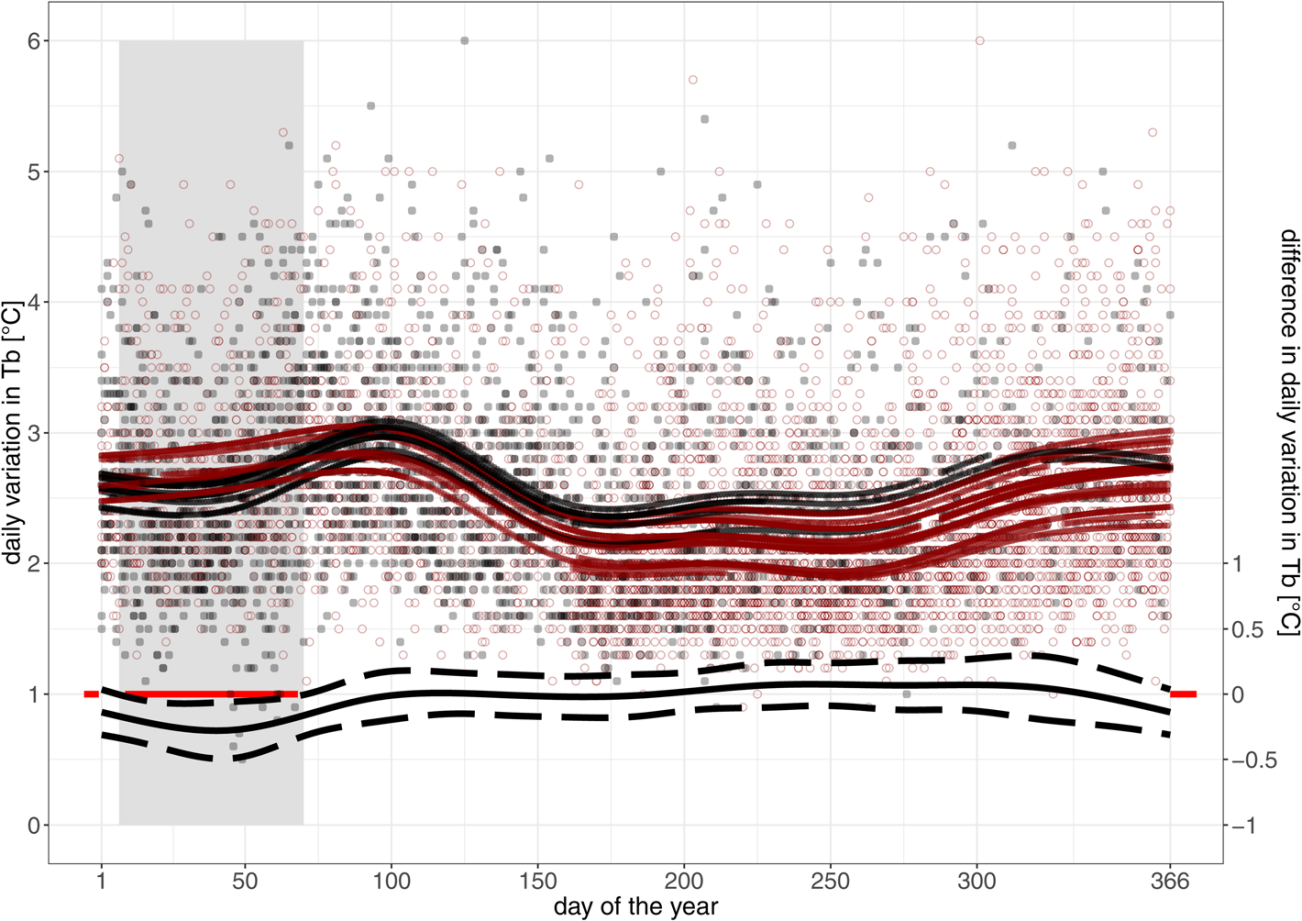
Daily variation in body temperature (T_b_) [°C, individual daily mamimum T_b_ – minimum *T*_*b*_] for eight pregnant (black hollow circles) and six non-pregnant wolverines (red hollow circles) over the year (day of the year 1 = 1st January). Fitted values from GAMM model are presented for pregnant (black lines) and non-pregnant individuals (red lines). On the bottom, the black line shows the average modelled difference in daily variation in T_b_ [°C] with 95% CI (dashed lines) between pregnant and non-pregnant wolverines, with a red line at 0 °C difference in the background as a reference level. The grey ribbon in the background shows the time span in which daily variation in T_b_ of pregnant females is significantly different from T_b_ of non-pregnant wolverines

Similar to daily mean T_b_, daily variation in T_b_ of pregnant and non-pregnant wolverines differed significantly from each other during the time of gestation, but showed similar patterns throughout the rest of the year. Pregnant females had significantly lower daily variation than non-pregnant individuals from DOY 7–70 with a modelled maximum difference of − 0.3 °C (95% CI: − 0.1 – − 0.5 °C) at DOY 40 (Fig. 4). Daily variation in T_b_ of non-pregnant females increased in the beginning of the year with a peak of 2.9 °C (95% CI: 2.8–3.0 °C) on DOY 85, followed by a steep decrease during summer with lowest value of 2.1 °C (95% CI: 2.0–2.2 °C) at DOY 251 and a second increase towards the end of the year (Fig. 4).

**Fig. 5:**
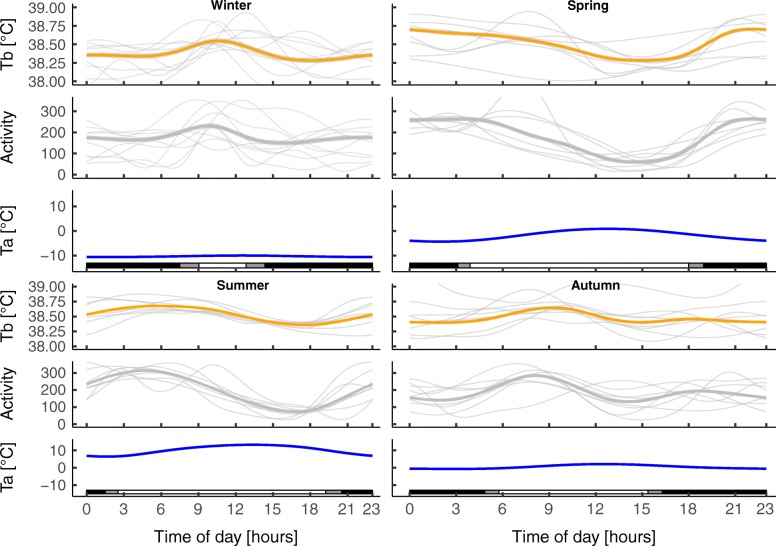
Seasonal patterns in body temperature (T_b_, [°C]) and activity patterns found in wolverines 2011–2014 in Sarek study area. Hourly and seasonal mean T_b_ (orange line), activity (grey line) and ambient temperature (T_a_, [°C], blue line) are presented with 95% CI in shaded grey, applying a cyclic cubic regression spline smoother for time of the day. Grey lines in the background represent individual hourly and seasonal mean values in T_b_ and activity. Changes in photoperiod are indicated at the bottom of each plot (black bar = darkness, grey bar = dawn/dusk, white bar = sunlight)

Daily patterns of both T_b_ and activity varied seasonally. In winter (December – February) wolverines showed an unimodal pattern in T_b._ Activity peaked around the same time as T_b_ but drops faster than T_b_ (Fig. 5, Winter). During this period there was, on average, only 2–4 h of daylight and little variation in T_a_ during the day and wolverines concentrated their activity between dawn and midday. In spring (March – May) wolverines were least active during the time of daylight with lowest values in the afternoon but were active on a constant level during night. T_b_ varied little but followed the same trend (Fig. 5, Spring). In summer (June – August) T_b_ and activity followed the same trend as in spring. Highest values could be detected just after sunrise, followed by a gradual decrease with lowest values in the late afternoon, which coincided with highest T_a_, followed by gradual increase towards the end of the day (Fig. 5, Summer). In autumn (September – November) wolverines showed a bimodal pattern in activity with highest values just after sunrise, followed by lowest values in late afternoon and another smaller peak just after sunset. T_b_ follows the same pattern with attenuated amplitude (Fig. 5, Autumn).

### Circadian rhythms in Tb and activity

**Fig. 6:**
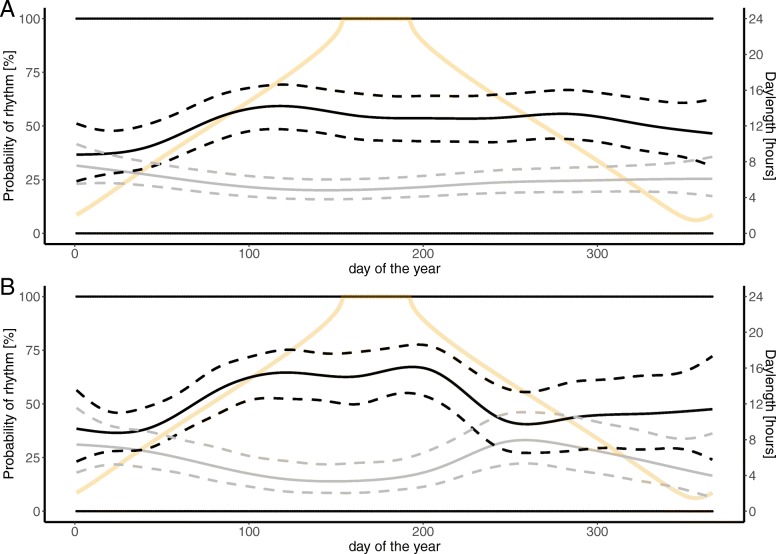
Prediction plots of probability of 24 h (black line) or 12 h (grey line) rhythm in body temperature (**a**) and activity (**b**) over the course of a year for wolverines, monitored from 2011 to 2014 in Sarek study area. Dashed lines represent 95% confidence interval, orange lines represent associated day length and black dots represent binary results of Lomb-Scargle Periodgram analysis (100 = rhythm present, 0 = rhythm absent). T_b_: *n* = 14 individuals, activity: *n* = 10 individuals

In total, 24 h circadian rhythms were present in 54 and 50.6% of the time for T_b_ and activity, respectively. Significant 12 h rhythms in T_b_ were present in 23.5% and in activity for 23.8% of the time. This means that the majority of rhythms displayed by wolverines over the year consisted of 24 and 12 h rhythms (T_b_: 77.5%, activity: 74.4%). The probability that wolverines displayed either of the two rhythms changed annually (Fig. 6). The highest probability that wolverines display a 24 h circadian rhythm in T_b_ was detected in April on DOY 111 with 61% [CI: 53–67%] (Fig. 6a). The highest probability for a 24 h circadian rhythm in activity was detected in July on DOY 194 with 67% [CI: 54–77%] (Fig. 6b). In January wolverines displayed the lowest probability of 24 h rhythm in both T_b_ (39% [CI: 32–47%]) and activity (36% [CI: 28–45%]). The probability of 12 h rhythms in both, T_b_ and activity followed an inverse trend of the 24 h rhythm and was generally lower (Fig. 6).

## Discussion

We showed that wolverine T_b_ varies over the year and is influenced by reproductive state. Reproducing females had significantly lower daily mean T_b_ and daily variation in T_b_ than non-reproducing females from early January to early March. Seasonally varying T_b_ and activity patterns suggest that generally wolverines are active both day and night, with peaks during crepuscular hours. The probability of 24 and 12 h rhythms in T_b_ and activity changes annually and is lowest in winter time.

Mean wolverine T_b_ was 38.5 ± 0.2 °C, which is comparable to previous observations of T_b_ in captive wolverines [30]. Wolverines showed a mean daily variation in T_b_ of 2.5 ± 0.7 °C which can be considered normal daily oscillations for mammals (1–4 °C; [3]). Maximum daily variations in T_b_ exceeded 4 °C occasionally in 60% of the individual months (Additional file 2), which indicates a high level of heterothermy. The ability to cope with cold temperatures by being locally heterothermic and applying peripheral cooling [59–61] is widely accepted but evidence for heterothermy of core T_b_ in circumpolar species is lacking. Increased heterothermy in wolverines could be caused by regular events of reduced T_b_, which in turn may be driven by an inadequate energy intake, as expected in cold environments [29]. Animals, which face inadequate energy intake may benefit from a reduced metabolic demand, accompanied by decreased T_b_. We observed the highest daily variation and lowest mean T_b_ from December – April (Additional file 2). A decrease from 38.5 °C to 35.8 °C T_b_ in March (Additional file 2) at an average T_a_ of − 10 °C represents a 5.5% energy saved (Additional file 4). This period is characterized by the lowest ambient temperatures and relatively low and unpredictable food availability for wolverines, as migrating semi-domestic reindeer, the main prey, return, from winter grazing lands in late April and early May [62]. The daily variation in T_b_ is suggested to provide an index of any compromise experienced by a free-ranging large mammal, reflecting response to challenging environmental conditions or effects of endogenous factors [29]. From November – April most our study area is snow covered and wolverines are exposed to increased risk of mortality, due to poaching facilitated by use of snowmobiles [40, 63]. During this period, wolverines select stronger for steep and rugged habitats that hinders snowmobile use [63, 64], indicating an influence of human disturbance on behaviour and habitat use. Furthermore, human disturbance can have direct effects on the physiology of individuals by a rise in T_b_, heart rate and/or metabolic rate [65–67]. The potential effect of human disturbance on both behaviour and physiology, suggests that snow season may represent the time of the year wolverines experience the biggest compromise, expressed by heterothermy.

Moreover, we found that the pattern of daily mean T_b_ of female wolverines differed between reproductive states, as reproductive females had significantly lower T_b_ from day 12–81 than non-reproducing females. Decreased T_b_ during gestation has been described in both domestic [20, 21] and wild mammals [5, 22, 68, 69]. Decreasing T_b_ during gestation in pregnant female brown bears is presumably caused by changes in progesterone levels, with maximum progesterone levels during implantation, followed by a decrease during gestation. Maintaining stable T_b_ during gestation and reducing maternal T_b_ may minimize hyperthermia events, which promotes fetal development [22]. Fetal T_b_ has been found to be dependent on and approximately 0.6 °C higher than maternal T_b_ [21]. Cell division stops at T_b_ higher than 40 °C [70], which makes a fetus potentially vulnerable to hyperthermia events [21].

Wolverines modified T_b_ and activity patterns over the year relative to photoperiod and ambient temperature. In general, activity was lowest in afternoon, similar to a previous study in the same area [71]. Furthermore, activity patterns changed with season, suggesting that wolverines may be active both day and night but with distinct peaks during crepuscular hours. Although changes in T_b_ were slightly delayed compared to changes in activity, both patterns were highly synchronized with peaks around the same time. The probability that wolverines exhibit a circadian rhythm in T_b_ and activity was lowest during polar nights and increased as the days became longer. This is contrary to our predictions that wolverines in northern Sweden will lose circadian rhythms in both polar night and day. Activity patterns in lynx (*Lynx lynx*) in northern Scandinavia were not influenced by daylight duration but rather by prey activity patterns [72] and circadian rhythms in sheep were disrupted due to environmental and dietary changes [73]. In late winter in northern ecosystems ungulate mortality increases due to starvation, avalanches or weakened animals [74]. Accordingly, wolverines are seasonally flexible in feeding strategy, shifting from mainly predation in spring and summer to scavenging in winter, as a response to increased carrion supply [75]. Changes in food availability feeding strategy may result in different activity patterns and attenuation of circadian rhythmicity in winter. Hence, photoperiod may not be the only influential factor, entraining circadian rhythms of wolverines in northern Sweden. Furthermore, circadian rhythmicity may also be influenced by a high individual component, which in turn may be influenced by e.g. demographic patterns and human disturbance.

## Conclusion

The use of combined biologging techniques shed new light into the physiology, activity patterns and reproductive biology of wolverines. This adds new information on a species, adapted to cold climates, which could not be obtained with other methods. Knowledge on ecophysiological characteristics of species are needed to address further questions of interest, such as potential vulnerability to effects of climate change and human encroachment.

## Additional files

Additional file 1: Graph to illustrate pregnancy determination based on daily mean body temperature and activity. (PNG 23 kb)

Additional file 2: Table of monthly averaged body temperature of non-pregnant and pregnant wolverines. (DOCX 13 kb)

Additional file 3: Autocorrelation plots of GAMM models. (DOCX 592 kb)

Additional file 4: Details about surgery and energy calculations. (DOCX 16 kb)

## Data Availability

Data used in this study are archived in the Dryad Digital Repository and will be accessible from one year following publication. Pending this time, data are available upon request from the wolverine project leader JP (jens.persson@slu.se).

## Abbreviations

CI: Confidence interval
DOY: Day of the year
GAMM: Generalised additive mixed effect model
GPS: Global positioning system
i.m.: Intramuscular
m.a.s.l: Meters above sea level
SD: Standard deviation
T_a_: Ambient temperature
T_b_: Body temperature
