# Supplementary figures and images for: Effects of reproduction and environmental factors on body temperature and activity patterns of wolverines
Source: Front Zool. 2019 Jun 17;16:21. doi: 10.1186/s12983-019-0319-8 (PMC6580505; doi:10.1186/s12983-019-0319-8)

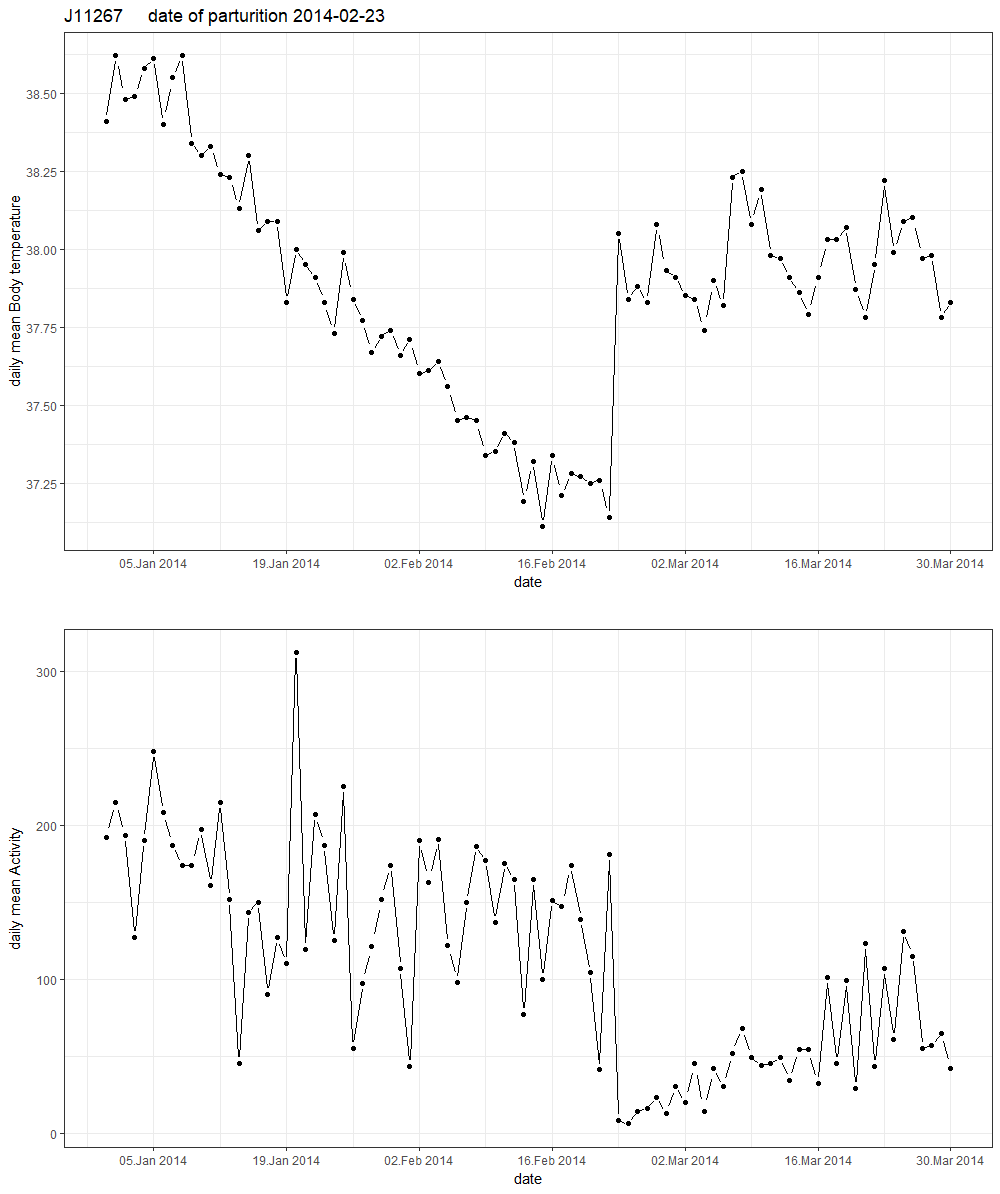

Supplement: Supplementary file 1 — Graph to illustrate pregnancy determination based on daily mean body temperature and activity. (PNG 23 kb) [file 12983_2019_319_MOESM1_ESM.png]
