# Supplementary material for: Effects of reproduction and environmental factors on body temperature and activity patterns of wolverines
Source: Front Zool. 2019 Jun 17;16:21. doi: 10.1186/s12983-019-0319-8 (PMC6580505; doi:10.1186/s12983-019-0319-8)
Supplement: Supplementary file 2 — Table of monthly averaged body temperature of non-pregnant and pregnant wolverines. (DOCX 13 kb) [file 12983_2019_319_MOESM2_ESM.docx]

**Table 1** Mean, minimum and maximum body temperature (*T_b_*, °C), standard deviation (SD) and daily range in *T_b_* (i.e. daily maximum *T_b_* - daily minimum *T_b_* for each individual) for each month for all 14 wolverines, monitored 2011-2014. Data from January – March are divided based on reproductive state (i.e. non-pregnant and pregnant). The male is included in the non-pregnant individuals. Sample size (n) is presented as individuals per month and individual year as well as number of unique individuals in brackets.

| Month | Reproductive state | Body temperature [°C] | | | | Daily range in individual body temperature [°C] | | | n |
| --- | --- | --- | --- | --- | --- | --- | --- | --- | --- |
|  |  | Mean | SD | Min | Max | Mean | Min | Max |  |
| Jan | Non-pregnant | 38.4 | 0.1 | 36.3 | 42.1 | 2.7 | 1.3 | 5.1 | 7 (6) |
|  | Pregnant | 38.2 | 0.1 | 35.4 | 42.1 | 2.6 | 1.1 | 5.0 | 10 (8) |
| Feb | Non-pregnant | 38.4 | 0.2 | 35.7 | 41.9 | 2.7 | 1.5 | 4.8 | 7 (6) |
|  | Pregnant | 37.8 | 0.2 | 34.7 | 41.4 | 2.5 | 0.9 | 4.5 | 10 (8) |
| Mar | Non-pregnant | 38.5 | 0.3 | 35.8 | 42.5 | 2.8 | 1.1 | 5.3 | 7 (6) |
|  | Pregnant | 38.2 | 0.2 | 35.2 | 41.5 | 2.9 | 0.8 | 5.1 | 10 (8) |
| Apr | All | 38.4 | 0.2 | 36.0 | 42.4 | 2.8 | 1.3 | 5.5 | 17 (12) |
| May | All | 38.5 | 0.3 | 35.5 | 42.0 | 2.5 | 1.1 | 5.0 | 16 (12) |
| Jun | All | 38.6 | 0.1 | 35.1 | 42.5 | 2.2 | 1.1 | 4.5 | 26 (14) |
| Jul | All | 38.6 | 0.2 | 35.1 | 42.6 | 2.2 | 1.0 | 5.8 | 24 (14) |
| Aug | All | 38.5 | 0.2 | 35.9 | 42.5 | 2.2 | 0.9 | 4.9 | 23 (14) |
| Sep | All | 38.5 | 0.1 | 36.1 | 42.5 | 2.2 | 0.9 | 4.8 | 22 (13) |
| Oct | All | 38.5 | 0.1 | 35.5 | 42.5 | 2.4 | 0.9 | 6.0 | 25 (13) |
| Nov | All | 38.5 | 0.1 | 36.4 | 42.4 | 2.5 | 0.9 | 5.2 | 21 (13) |
| Dec | All | 38.5 | 0.1 | 35.9 | 42.3 | 2.6 | 1.2 | 5.3 | 19 (13) |
