# Supplementary material for: Effects of reproduction and environmental factors on body temperature and activity patterns of wolverines
Source: Front Zool. 2019 Jun 17;16:21. doi: 10.1186/s12983-019-0319-8 (PMC6580505; doi:10.1186/s12983-019-0319-8)

Figur 1: Autocorrelation plot of Generalised additive mixed model on individual daily mean body temperature of 14 wolverines, monitored from 2011 – 2014 in Sarek study area.


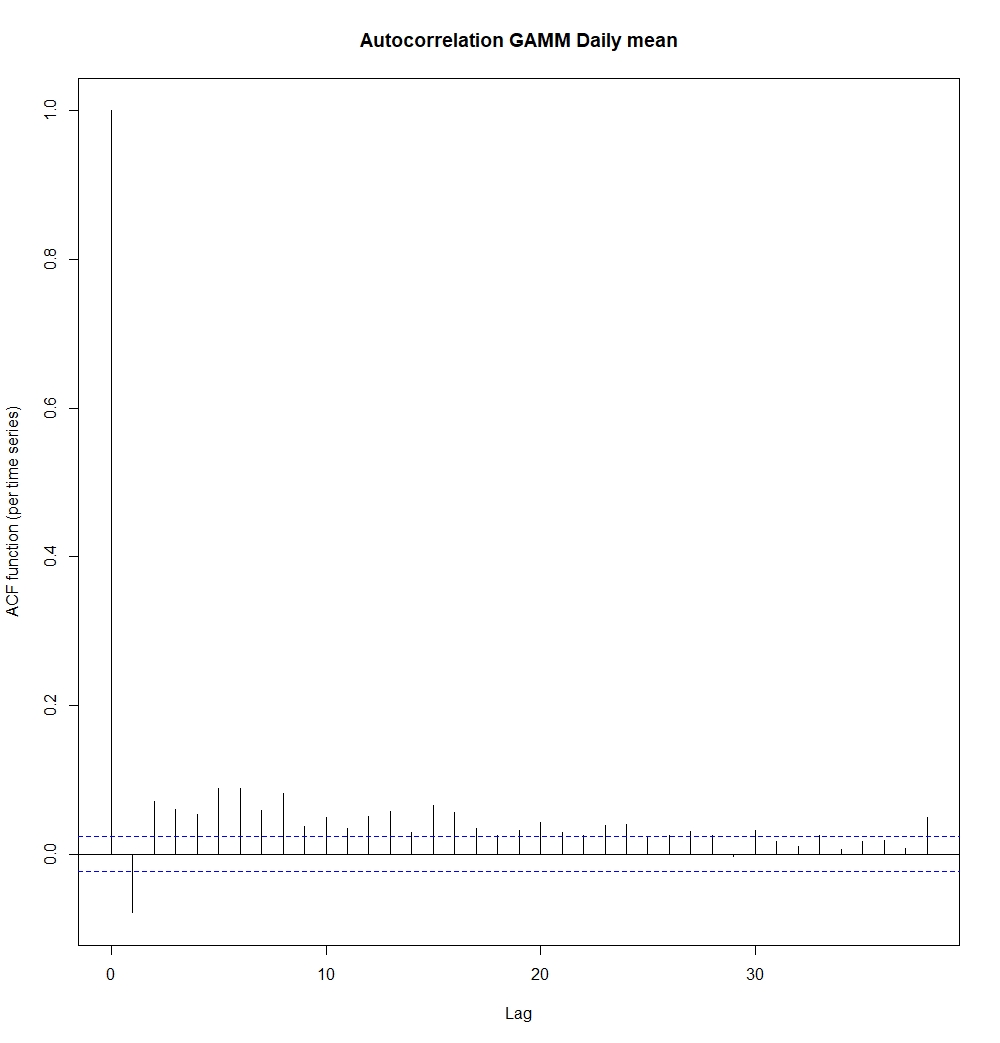


Figur 2: Autocorrelaton plot of Generalised additive mixed effect model on individual daily variation in body temperature of 14 wolverines, monitored from 2011 – 2014 in Sarek study area.


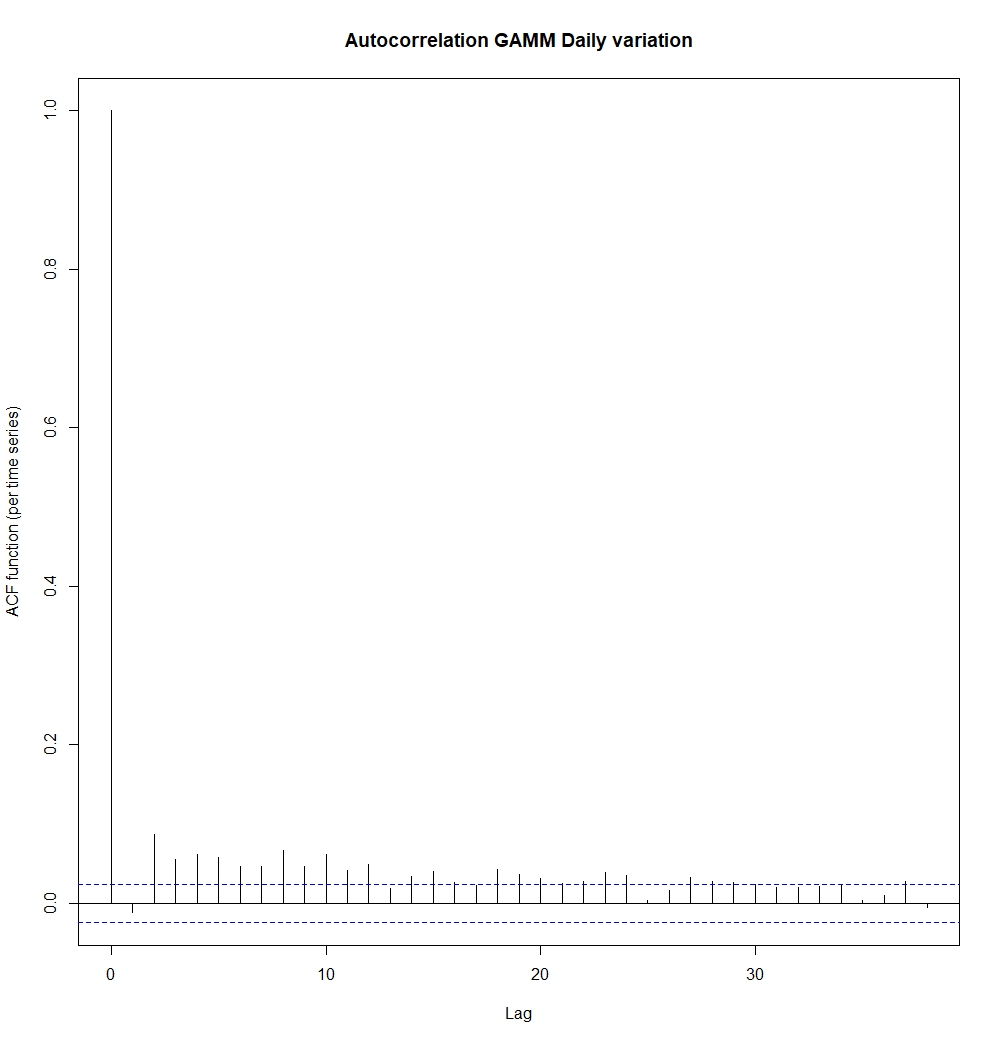


Figur 3: Autocorrelation plot of Generalised additive mixed model on 24 hours circadian rhythm in body temperature of 14 wolverines, monitored from 2011 – 2014 in Sarek study area.


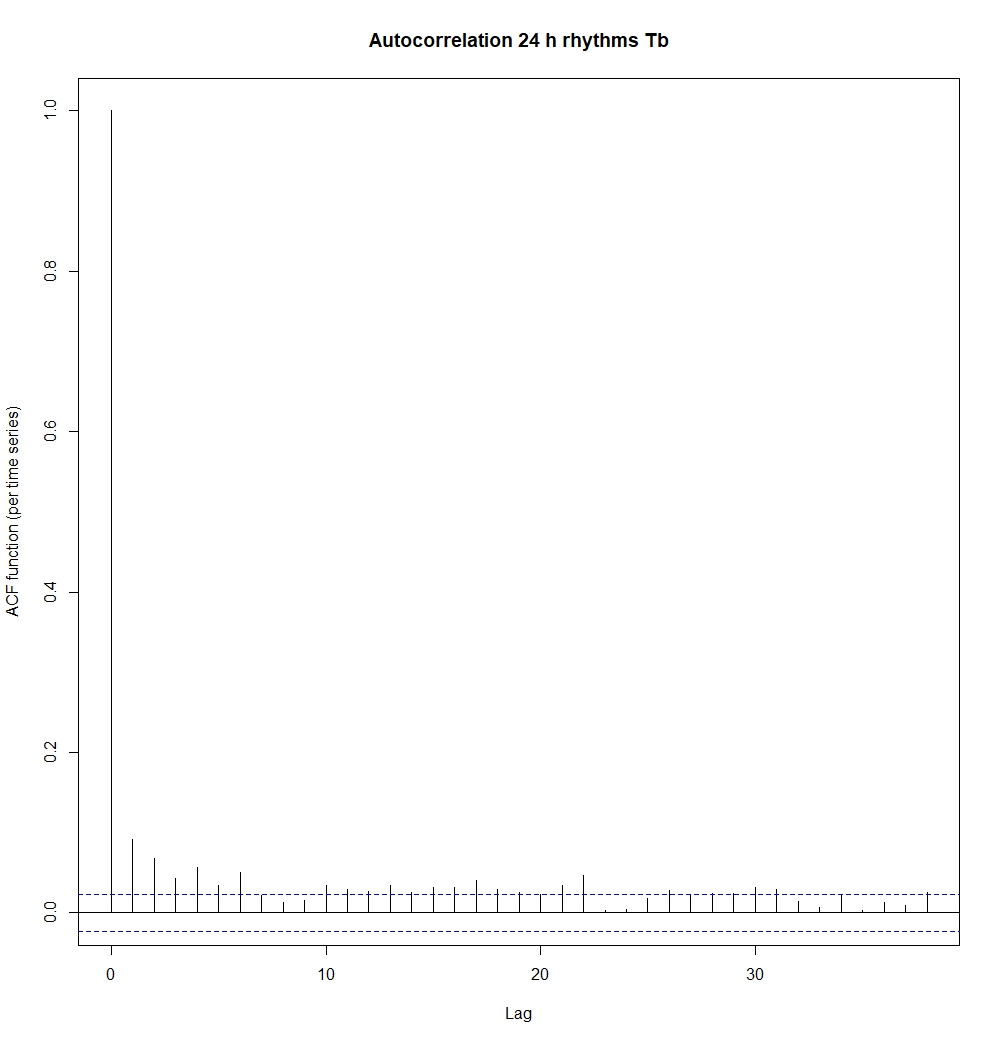


Figur 4: Autocorrelation plot of Generalised additive mixed model on 12 hours rhythm in body temperature of 14 wolverines, monitored from 2011 – 2014 in Sarek study area.


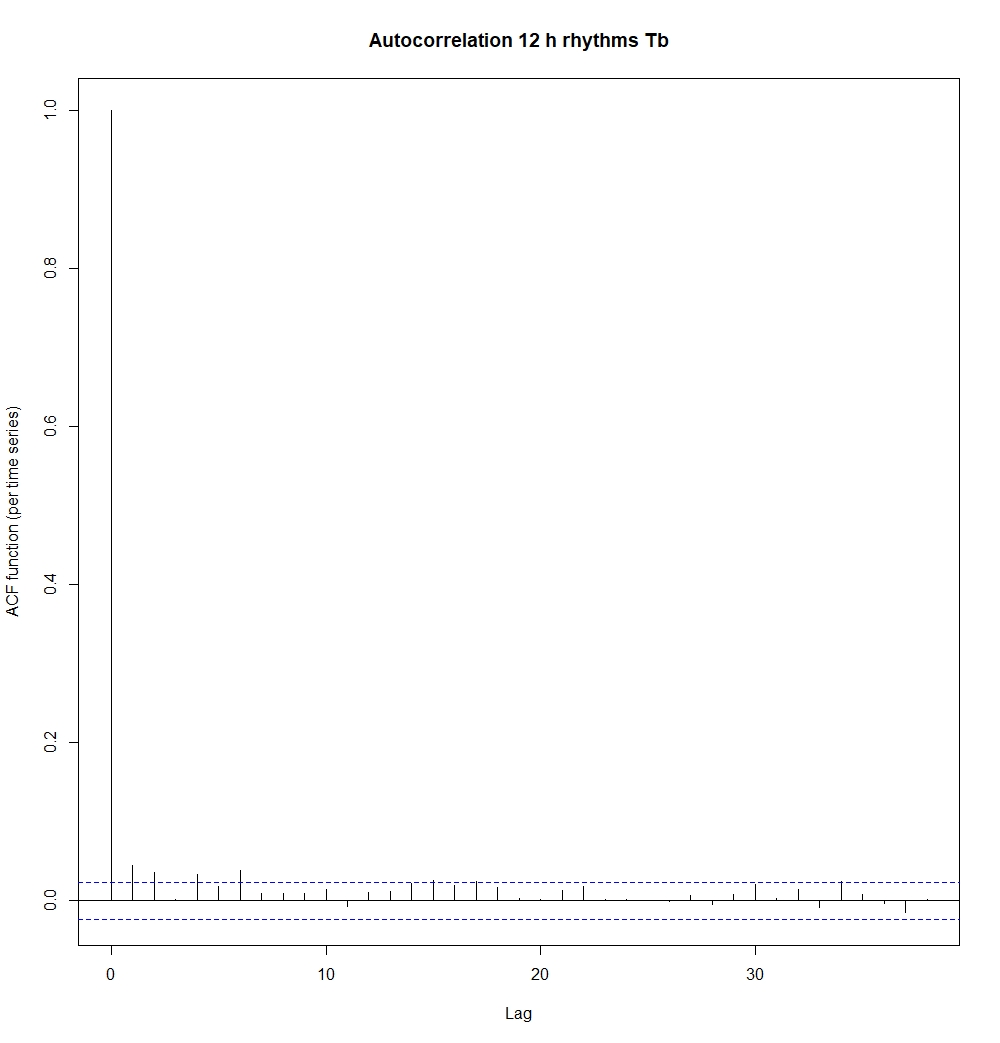


Figur 5: Autocorrelation plot of Generalised additive mixed model on 24 hours circadian rhythm in activity of 10 wolverines, monitored from 2011 – 2014 in Sarek study area.


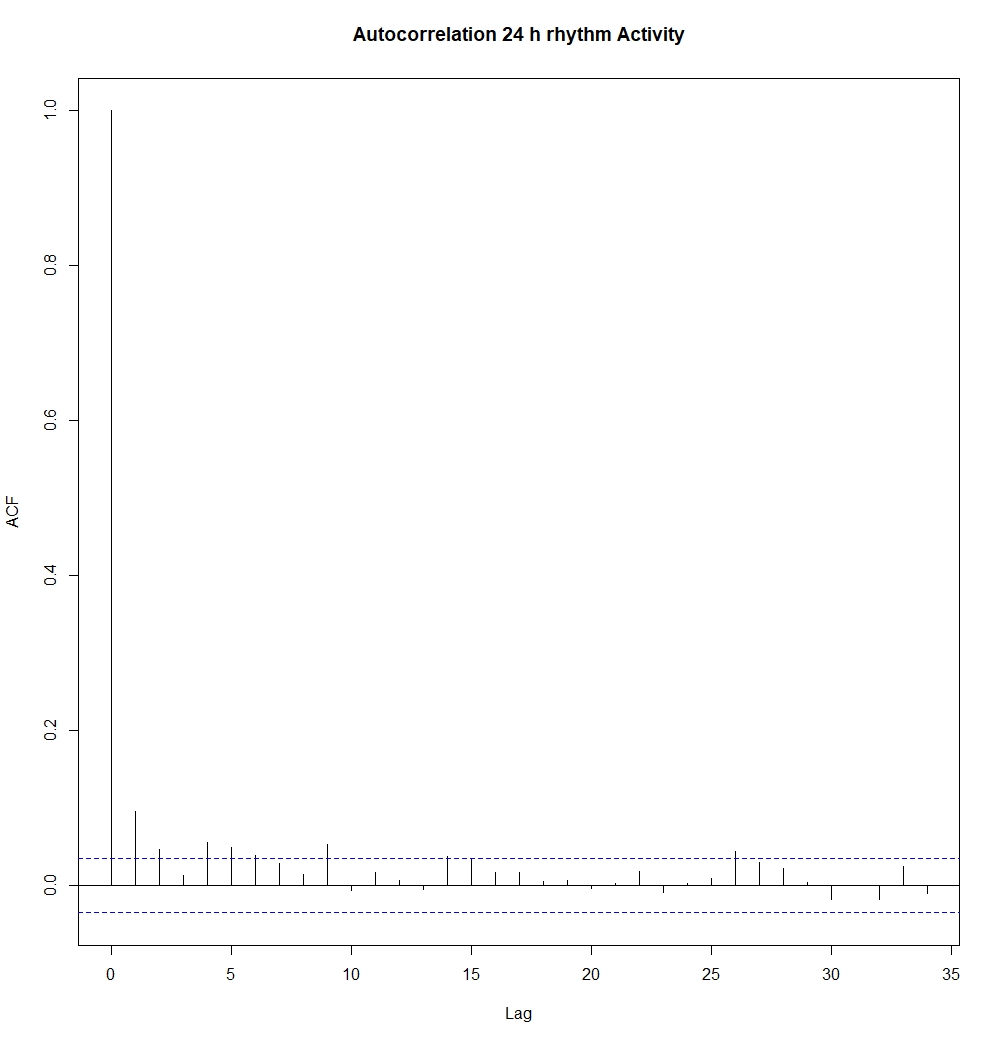


Figur 6: Autocorrelation plot of Generalised additive mixed model on 12 hours rhythm in activity of 10 wolverines, monitored from 2011 – 2014 in Sarek study area.


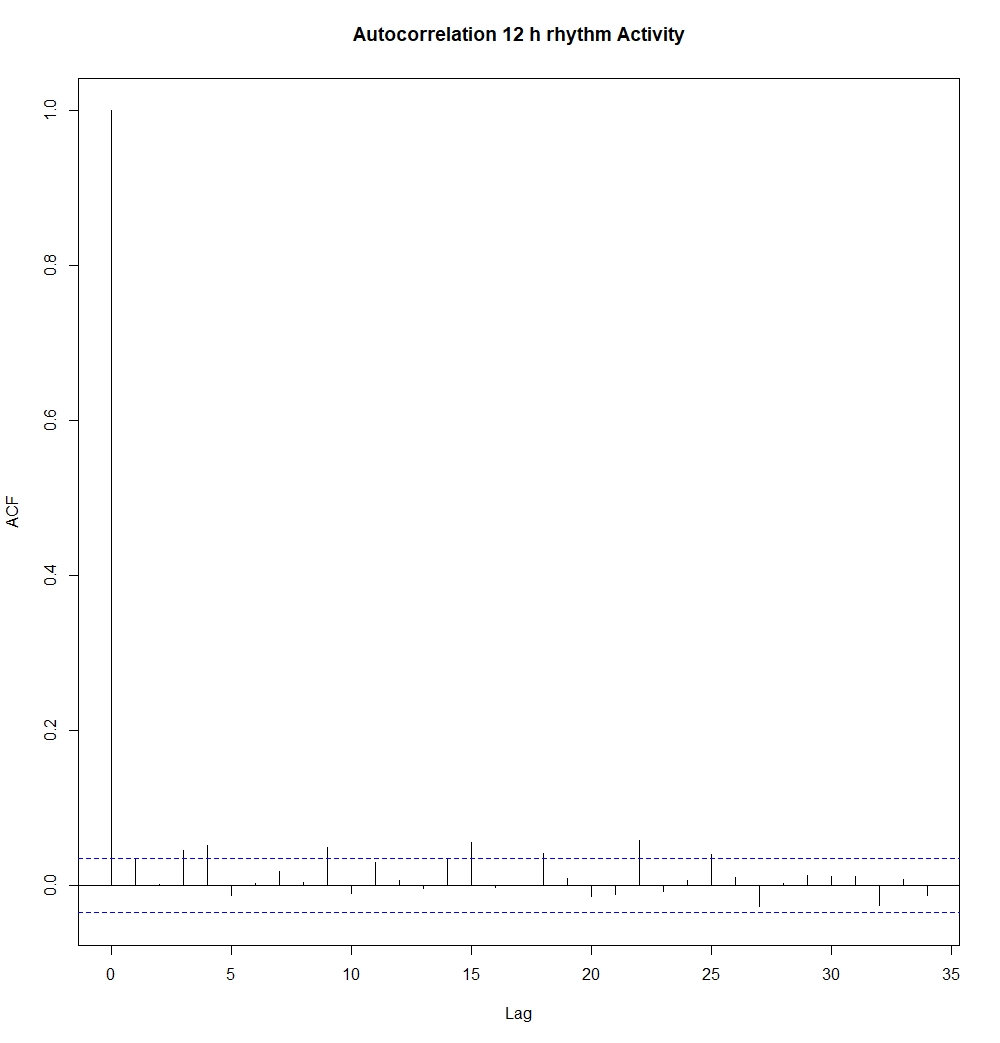


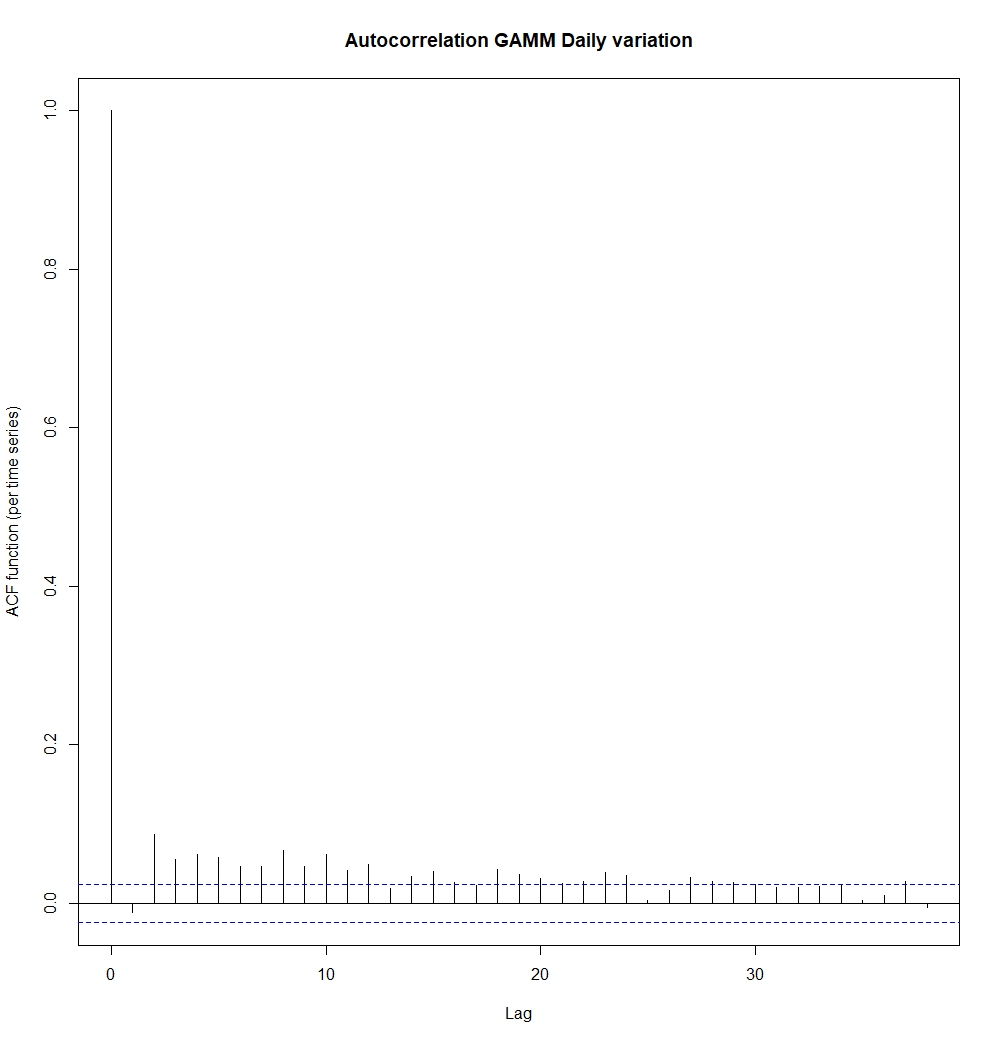

Supplement: Supplementary file 3 — Autocorrelation plots of GAMM models. (DOCX 592 kb) [file 12983_2019_319_MOESM3_ESM.docx]
