# Supplementary material for: Effects of reproduction and environmental factors on body temperature and activity patterns of wolverines
Source: Front Zool. 2019 Jun 17;16:21. doi: 10.1186/s12983-019-0319-8 (PMC6580505; doi:10.1186/s12983-019-0319-8)
Supplement: Supplementary file 4 — Details about surgery and energy calculations. (DOCX 16 kb) [file 12983_2019_319_MOESM4_ESM.docx]

**Surgery details**

Temperature loggers were sterilized using ethylene oxide gas. For post-operative analgesia, 0.2 mg/kg meloxicam (Metacam®, Boehringer Ingelheim, Germany) was administered subcutaneously. For surgery, the animals were kept in dorsal recumbency. An appropriate area caudal to the umbilicus was clipped and swabbed with chlorhexidine in 60% ethyl alcohol (Klorhexidin®, Fresenius Kabi Norge AS, Halden, Norway). For access to the peritoneal cavity, a ventral midline incision was made using standard surgical procedures and the temperature logger was placed free-floating in the peritoneal cavity. The incision was closed in two layers with absorbable sutures (PDS®, Johnsen & Johnsen, Diegem, Belgium) and the skin wound was covered with a spray dressing (Opsite®, Smith & Nephew Medical Ltd, Hull, England).

**Calculations of energy savings**

Percentage of energy saved by reducing T_b_ was based on the following formula:

$$Energy saved=100-(\frac{MR2}{MR1}*100)$$

MR1 represents metabolic rate at mean T_b_ in March (38.5°C) at on average -10°C ambient temperature and MR2 represents metabolic rate when Tb is reduced to (35.8°C) at the same ambient temperature. Calculation based on Heldmaier and Ruf [1]

$$MR1=C*\left( Tb-\mathrm{Ta} \right)$$

Thermal conductance ( C) is calculated for a wolverine of 10 kg based on Aschoff [2]

$$\log\left( C \right)=\log\left( 0.844 \right)-0.462*\log(10000 g)$$

**References**

1. Heldmaier G, Ruf T. Body temperature and metabolic rate during natural hypothermia in endotherms. Journal of Comparative Physiology B-Biochemical Systems and Environmental Physiology. 1992;162(8):696-706.

2. Aschoff J. Thermal conductance in mammals and birds - its dependence on body size and circadian phase. Comp Biochem Physiol A-Mol Integr Physiol. 1981;69(4):611-9.
